# Rad53 homologue forkhead-associated kinase A (FhkA) and Ca^2+^-binding protein 4a (CBP4a) are nucleolar proteins that differentially redistribute during mitosis in *Dictyostelium*

**DOI:** 10.1186/1747-1028-8-4

**Published:** 2013-04-12

**Authors:** Andrew Catalano, Danton H O’Day

**Affiliations:** 1 Department of Cell and Systems Biology, University of Toronto, 25 Harbord st., Toronto, ON M5S 3G5, Canada; 2 Department of Biology, University of Toronto at Mississauga, 3359 Mississauga rd. N., Mississauga, ON L5L 1C6, Canada; 3 Department of Chemistry, City College of New York, 160 Convent Ave., New York, NY 10031, USA

**Keywords:** Forkhead-associated kinase A, Ca^2+^-binding protein 4a, Nucleolus, Mitosis, Actinomycin-D, *Dictyostelium*, Cell cycle

## Abstract

**Background:**

During mitosis most nucleolar proteins redistribute to other locales providing an opportunity to study the relationship between nucleolar protein localization and function. *Dictyostelium* is a model organism for the study of several fundamental biological processes and human diseases but only two nucleolar proteins have been studied during mitosis: NumA1 and Snf12. Both of them are linked to the cell cycle. To acquire a better understanding of nucleolar protein localization and dynamics in *Dictyostelium* we studied the nucleolar localization of two additional proteins during mitosis: Snf12-linked forkhead-associated kinase A (FhkA), which is involved in the cell cycle, and Ca^2+^-binding protein 4a (CBP4a), which is a binding partner of NumA1.

**Methods:**

Polyclonal antibodies were produced in-house. Cells were fixed and probed with either anti-FhkA or anti-CBP4a in order to determine cellular localization during interphase and throughout the stages of mitosis. Colocalization with DAPI nuclear stain allowed us to determine the location of the nucleus and nucleolus while colocalization with anti-α-tubulin allowed us to determine the cell cycle stage.

**Results:**

Here we verify two novel nucleolar proteins, Rad53 homologue FhkA which localized around the edge of the nucleolus and CBP4a which was detected throughout the entire nucleolus. Treatment with the Ca^2+^ chelator BAPTA (5 mM) showed that the nucleolar localization of CBP4a is Ca^2+^-dependent. In response to actinomycin D (0.05 mg/mL) CBP4a disappeared from the nucleolus while FhkA protruded from the nucleus, eventually pinching off as cytoplasmic circles. FhkA and CBP4a redistributed differently during mitosis. FhkA redistributed throughout the entire cell and at the nuclear envelope region from prometaphase through telophase. In contrast, during prometaphase CBP4a relocated to many large, discrete “CBP4a islands” throughout the nucleoplasm. Two larger “CBP4a islands” were also detected specifically at the metaphase plate region.

**Conclusions:**

FhkA and CBP4a represent the sixth and seventh nucleolar proteins that have been verified to date in *Dictyostelium* and the third and fourth studied during mitosis. The protein-specific distributions of all of these nucleolar proteins during interphase and mitosis provide unique insight into nucleolar protein dynamics in this model organism setting the stage for future work.

## Background

The nucleolus is no longer considered to be just a ribosomal factory. It is now recognized as a multifunctional nuclear subcompartment involved in a multitude of biological processes and human diseases [1-8]. During mitosis the nucleolus disassembles and nucleolar proteins redistribute to non-nucleolar locales [9-12]. This change in localization may be linked to changes in function which may explain why some nucleolar proteins undergo different phosphorylation events during mitosis than during interphase [13-15]. Considering the resurgence of interest in the nucleolus and its central role in various diseases and cell stress, finding appropriate genetically-tractable model systems in order to study the relationship between nucleolar protein localization, cell cycle, and function is an important goal [3].

*Dictyostelium* is a model eukaryote for the study of several fundamental biological processes as well as several human diseases however little is known about its nucleolus and even less is known about the nucleolar events that occur during the closed mitosis that occurs in this organism [16-18]. The *Dictyostelium* nucleolus is different from that of most organisms in that it is composed of 2–4 patches adjacent to the inner nuclear membrane as opposed to being a single entity located free within the nucleoplasm [19-21]. Of five nucleolar proteins identified to date, only two have been studied during mitosis: the calmodulin (CaM)-binding protein nucleomorphin (NumA1) and BAF60a homologue Snf12 [22-28]. NumA1 redistributes to discrete, unidentified nuclear subdomains during mitosis while Snf12 redistributes throughout the entire cell, despite the intact nuclear envelope that remains during mitosis in *Dictyostelium*[24,25].

We therefore set out to identify novel cell cycle-linked nucleolar proteins in *Dictyostelium* and to investigate their dynamics during mitosis in order to better understand the relationship between nucleolar protein localization and dynamics during the cell cycle in this model eukaryote. To identify such proteins, we examined those linked to either Snf12 or NumA1; the only nucleolar proteins in *Dictyostelium* known to undergo mitotic redistribution. Snf12 possesses a SWIB/MDM2 domain which in higher eukaryotes is also found in the cell cycle regulator MDM2 [25]. MDM2 interacts with DNA damage response protein Chk2 (Rad53 in yeast) suggesting that *Dictyostelium* Chk2/Rad53 homologue forkhead-associated kinase A (FhkA) could reside within the nucleolus with Snf12 and may also have ties to the cell cycle [29-33]. In higher eukaryotes Chk2 (Rad53 in yeast) responds to DNA damage by activating several downstream effectors such as p53 and BRCA1 which eventually leads to cell cycle arrest [29,33]. *Dictyostelium* FhkA may therefore also be involved in such cell cycle checkpoint events and is therefore a good candidate for choosing nucleolar proteins linked to the cell cycle in *Dictyostelium*. Unlike most other organisms, *Dictyostelium* possesses five Chk2/Rad53 homologues: FhkA, B, C, D, and E. It is not known why all five are needed however we have chosen FhkA because of the five *Dictyostelium* homologues its sequence is most similar to Rad53.

NumA1 localizes predominately to nucleoli but is also present in the nucleoplasm [24,27]. NumA1 likely interacts with binding-partner puromycin-sensitive aminopeptidase A (PsaA) in the nucleoplasm, since this is where the two colocalize, however its nucleolar binding partner has yet to be identified [34,35]. The only other known NumA1-interacting protein is Ca^2+^-binding protein 4a (CBP4a) suggesting it may also reside in the nucleolus [36]. CBP4a is one of 13 Ca^2+^-binding proteins (CBPs) in *Dictyostelium* and although its function remains unknown its interaction with NumA1 suggests that it may be involved in cell cycle events [36-40]. This is based on several lines of evidence linking NumA1 to the cell cycle such as its interaction with PsaA, a protein involved in cell cycle events, and the presence of several protein domains which are found in cell cycle proteins [27,34,41-43]. Recent studies on *Dictyostelium* PsaA localization and binding-proteins support its putative role in the cell cycle [42,43]. CBP4a is almost identical in sequence to *Dictyostelium* CBP4b, another Ca^2+^-binding protein whose function is unknown, but not similar to any other protein from any organism.

The work presented here revealed that FhkA and CBP4a each localized to different regions of interphase nucleoli. During mitosis, FhkA redistributed throughout the entire cell while CBP4a redistributed to nuclear areas which were different from those observed for NumA1. The unique pattern of CBP4a localization during mitosis suggests there may be previously unidentified intranuclear subdomains linked to mitosis in *Dictyostelium*. For comparative purposes, we also investigated the redistribution of FhkA and CBP4a in response to treatment with actinomycin-D (AM-D), which is known to result in the redistribution of several nucleolar proteins in *Dictyostelium* as well as in other organisms [22,24,25]. In response to AM-D treatment both FhkA and CBP4a exhibited a response similar to that observed for Snf12 [25]. In total the results presented here yield new insight into nucleolar protein dynamics in *Dictyostelium* and suggest that previously unidentified intranuclear domains exist during mitosis.

## Results

### Sequence analysis of FhkA

**Figure 1:**
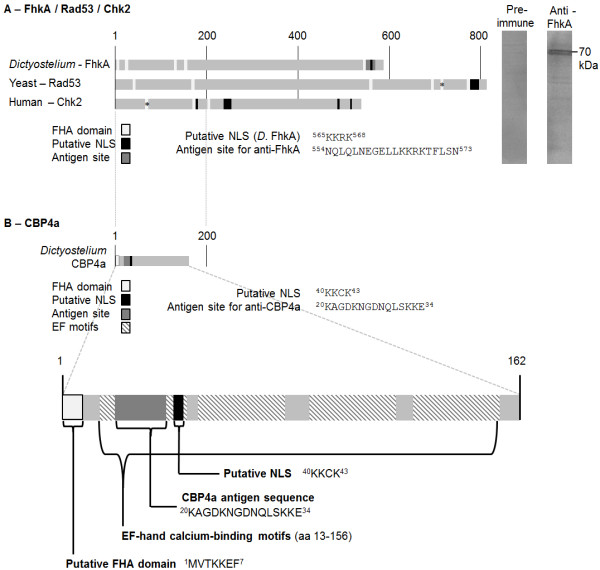
**Domain structure diagram of FhkA and CBP4a. (A)** FhkA (Rad53 in yeast, Chk2 in humans) possesses several putative FHA domains (asterisk indicates verified domains [47,48]). Putative NLSs and the antigen site for anti-FhkA are indicated. Numbers represent amino acid position. Western blot shows the detection of FhkA by anti-FhkA at ~70 kDa as well as the absence of FhkA detection by the preimmune serum. **(B)** CBP4a possesses a putative FHA domain and putative NLS as well as four EF-hand Ca^2+^-binding motifs. The antigen site is shown for both FhkA and CBP4a.

A ClustalW2 alignment showed that *Dictyostelium* FhkA is 28.8% identical and 53.6% conserved to yeast Rad53 and 27.8% identical and 45.7% conserved to human Chk2. It possesses several putative forkhead-associated (FHA) domains common to nuclear proteins and a putative NLS (Figure 1A) [44,45]. Rad53 and Chk2 also possess multiple FHA domains some of which have been verified [46-48].

A BLAST search revealed Rad53, Chk2, and FhkA all possess domains characteristic of the FHA superfamily of proteins as well as putative phosphopeptide binding sites, an ATP-binding domain, and an activation domain. These regions display higher levels of homology than the rest of the protein sequences. It is important to note that *Dictyostelium* possesses five Rad53 family members, FhkA, B, C, D, and E, the significance of which is unknown [46].

### Sequence analysis of CBP4a

Analysis of CBP4a via the Eukaryotic Linear Motif database also revealed the presence of a putative FHA domain, ^1^MVTKKEF^7^ (Figure 1B) [45]. CBP4a also contains four EF-hand Ca^2+^-binding motifs (EF-hand motif) and a putative NLS in its C-terminal (Figure 1B). CBP4a is identical to *Dictyostelium* CBP4b except for one residue. The function of CBP4b is also unknown. A BLAST search revealed that other than CBP4b, CBP4a is not similar in sequence to any other protein.

### FhkA localizes to the nucleolar periphery

To determine the localization of FhkA, immunolocalization was performed using anti-FhkA. Western blots of cell lysates were probed with anti-FhkA to determine the specificity of this antibody. Anti-FhkA detected a band ~70 kDa corresponding to the predicted molecular weight of FhkA (66 kDa) demonstrating this antibody specificity detects FhkA (Figure 1A). Conversely, western blots probed with preimmune serum did not reveal any significant bands. Anti-FhkA was therefore used for all immunolocalization experiments.

**Figure 2:**
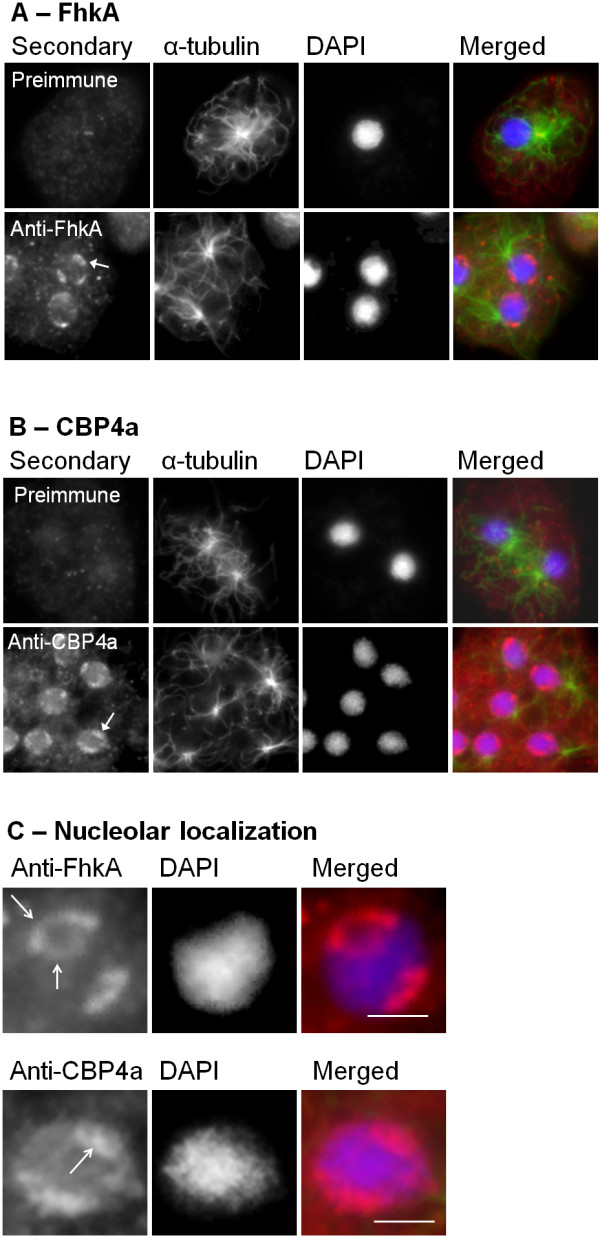
**Localization of FhkA and CBP4a. (A)** Unlike for cells probed with preimmune serum, which displayed diffuse/weak fluorescence, anti-FhkA detected FhkA in untreated cells at the edge of nucleolar patches as demonstrated by colocalization with DAPI counterstain **(B)** Similarly, cells probed with anti-CBP4a preimmune serum also displayed diffuse/weak fluorescence however anti-CBP4a detected CBP4a in nucleolar patches as demonstrated by colocalization with DAPI counterstain. Merged image displays overlay of FhkA (red), α-tubulin (green), and DAPI (blue). **(C)** Enlarged view of nuclei showing nucleolar localization pattern of FhkA and CBP4a. Nucleoli are indicated (arrows). At least four independent replicates were performed, all of which showed the same result. Scale bar represents 2 μm.

FhkA was detected by anti-FhkA at the periphery of the nucleolar patches, as demonstrated by colocalization with the unstained DAPI regions of the nucleus (Figure 2A, C). Within each patch FhkA was detected predominately at the periphery adjacent to the nuclear envelope and to a lesser extent, the periphery adjacent to the nucleoplasm. In contrast, cells that were probed with preimmune serum in place of anti-FhkA displayed diffuse fluorescence throughout the entire cell (Figure 2A).

### CBP4a localizes Ca^2+^-dependently to nucleoli

To determine the localization of CBP4a immunolocalization was performed using anti-CBP4a, the specificity of which had previously been verified via western blots [49]. CBP4a was detected in the nucleolus by anti-CBP4a however unlike FhkA, CBP4a was detected within the body of the nucleolar patches (Figure 2B, C). As well, cells probed with pre-immune serum in place of anti-CBP4a displayed a diffuse pattern of fluorescence throughout the entire cell with slightly greater, but still uniform, intensity in the nucleus (Figure 2B).

### AM-D treatment causes nucleolar protrusion and differential redistribution of FhkA to cytoplasmic circles

Treatment with AM-D, an inhibitor of transcription in other organisms, has previously been shown to lead to nucleolar dissolution and the disappearance of nucleolar proteins NumA1 and eukaryotic initiation factor 6 (eif6) from the nucleolus in *Dictyostelium*[20,22,24]. In stark contrast, AM-D induces the translocation of nucleoplasmic Snf12 to nucleoli followed by the protrusion of nucleolar Snf12 from the nucleus, a phenomenon not previously observed in any organism [25]. Since FhkA is a resident nucleolar protein we were interested to determine what effect AM-D might have on its localization.

**Figure 3:**
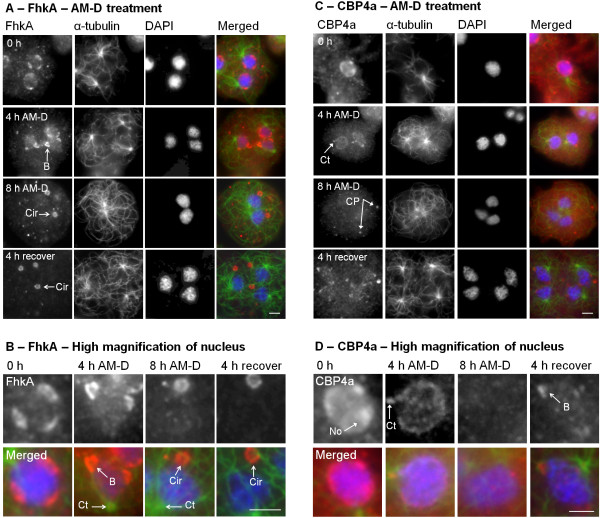
**Effect of AM-D on FhkA and CBP4a localization. (A)** AM-D treatment (4 h) resulted in the budding of nucleolar-localized FhkA from the nucleus. After 8 hours FhkA was detected as cytoplasmic circles adjacent to the nucleus, always on the opposite side as the centrosome. After recovery (4 hr) from AM-D treatment FhkA was still detected in cytoplasmic circles (Cir) which were not adjacent to the nucleus. **(B)** Enlargement of nucleus from **(A)**. **(C)** In AM-D-treated cells (4 hr) the intranuclear patches detected by anti-CBP4a were diminished in size compared to those detected in untreated cells. These patches ultimately disappeared after 8 hr treatment. **(D)** Enlargement of nucleus from **(C)**. Four hour treatment also resulted in detection of CBP4a in the centrosomal region (Ct) while 8 hour treatment resulted in the presence of cytoplasmic particles (CP). Treatment for 4 hours followed by a 4 hour recovery period resulted in only a partial restoration of nucleoplasmic CBP4a. Scale bar represents 2 μm. CBP4a budding (B), cytoplasmic particles (CP), centrosomes (Ct), and nucleoli (No) are indicated. At least four independent replicates were performed, all of which showed the same result.

After 4 hours of AM-D treatment FhkA was detected along the periphery of nucleolar patches which were protruding from the nucleus, similar to the response observed for Snf12 (Figure 3A, B). FhkA was detected predominately at the periphery of the protruding body and to a lesser degree in the region joining the protrusion to the nucleoplasm. After 8 hours of treatment these protrusions were no longer visible, however FhkA was instead detected in cytoplasmic circles adjacent to, and apparently connected to, the nucleus (Figure 3A, B). Similar to the protrusions, FhkA was detected around the periphery of each cytoplasmic circle but was relatively absent from the region joining the circle to the nucleus. After recovery from AM-D treatment FhkA was not detected in the nucleus or nucleolus but rather remained in cytoplasmic circles that were now in close proximity to, but apparently detached from, the nucleus (Figure 3A, B). It is interesting to note that the protrusions and cytoplasmic circles were almost always detected in regions of the nucleus distant from the centrosome (Figure 3A, B). These results are remarkably similar to those observed for Snf12 [25].

### AM-D treatment causes nucleolar protrusion and differential redistribution of CBP4a to cytoplasmic circles

AM-D treatment had a somewhat different effect on CBP4a localization. After 4 hours of treatment CBP4a was not detected in nucleoli and no protrusions were observed, similar to that observed for NumA1 and eif6 (Figure 3C, D) [22,24]. Although absent from nucleoli, CBP4a was still present in the nucleoplasm, to a degree. However after 8 hours CBP4a was still absent from nucleoli but detected as cytoplasmic circles, much smaller than the FhkA circles. After 4 hours treatment followed by 4 hours of recovery CBP4a was detected in nucleolar patches appearing to protrude from the nucleus, similar to that observed for FhkA (Figure 3C, D).

### AM-D-induced nucleolar protrusions are not dependent on associated microtubules

**Figure 4:**
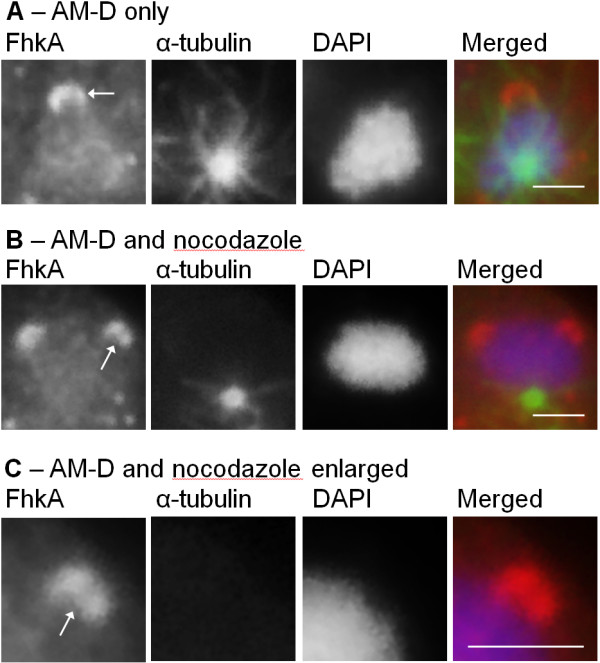
**Nucleolar protrusions are not dependent on microtubules. (A)** Nucleus of cell treated with AM-D for 4 hours. **(B)** Nucleus of cell treated with both AM-D and nocodazole for 4 hours. **(C)** Enlarged view of protrusion from **(B)**. A merged image shows the overlay of FhkA (red), α-tubulin (green), and DAPI (blue). Scale bar represents 2 μm. At least four independent replicates were performed, all of which showed the same result.

The nucleolar protrusions observed after AM-D treatment are similar in morphology to the nozzle-like nucleolus of aggregating *Dictyostelium* cells which also protrudes from the nucleus [50]. The position and orientation of these nozzle-like structures are dependent on adjacent microtubules [50]. We were therefore interested to determine if the AM-D-induced nucleolar protrusions are also dependent on microtubules. Colocalization of the nucleolar protrusions with microtubules suggests there is no association between the two however it is difficult to be sure due to the high number of microtubules present (Figure 4A). Cells were therefore treated with both AM-D (0.05 mg/mL, 4 hours) as well as the microtubule inhibitor nocodazole (50 μM, 4 hours) and probed with anti-FhkA. It is clear that in the absence of microtubules the nucleolar protrusions are still detected (Figure 4B). Moreover, the protrusions still occur in regions distant from the centrosome. These results demonstrate that the existence and position of the nucleolar protrusions are not dependent on associated microtubules.

### FhkA redistributes throughout the cytoplasm during mitosis

**Figure 5:**
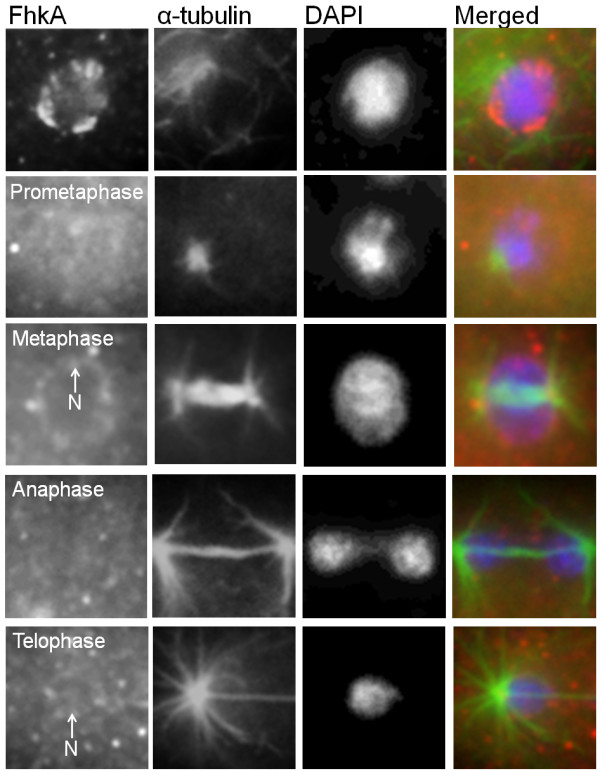
**FhkA redistributes throughout the cytoplasm during mitosis.** The pattern of microtubules (α-tubulin) was used to determine the stage of mitosis and DAPI was used to identify the nucleus. During prometaphase through telophase FhkA was detected throughout the entire cell. It was also detected at the nuclear envelope region (N). A merged image for each stage shows the overlay of FhkA (red), α-tubulin (green), and DAPI (blue). At least four independent replicates were performed, all of which showed the same result. Scale bar represents 2 μm.

A common feature of mammalian nucleolar proteins is their redistribution to non-nucleolar locales during mitosis [9-12]. Recently, *Dictyostelium* nucleolar proteins NumA1 and Snf12 have also been shown to redistribute differentially during mitosis [24,25]. We therefore examined whether FhkA undergoes a similar redistribution. During prometaphase and metaphase FhkA was detected by anti-FhkA throughout the entire cell as well as at the nuclear envelope region (Figure 5). During anaphase it was detected throughout the entire cell but no longer at the nuclear envelope region. During telophase however, FhkA was detected throughout the entire cell and at the nuclear envelope region (Figure 5).

### CBP4a redistributes to intranuclear regions during mitosis

**Figure 6:**
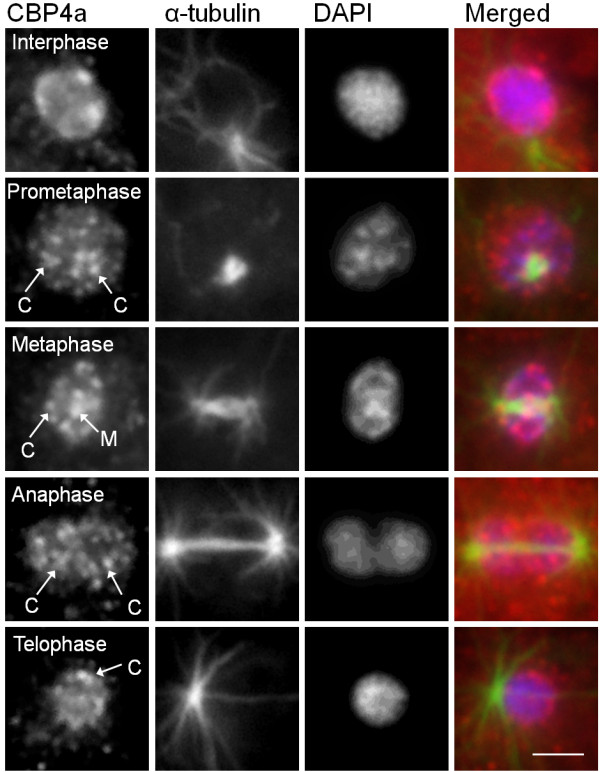
**CBP4a redistributes to intranuclear “CBP4a islands” during mitosis.** The pattern of microtubules (α-tubulin) was used to determine the stage of mitosis and DAPI was used to identify the nucleus. CBP4a localized to discrete “CBP4a islands” (C) throughout the nucleoplasm during prometaphase through telophase, at least two larger “CBP4a islands” at the metaphase plate region (M) during metaphase, and large “CBP4a islands” around the inner nuclear membrane during telophase. A merged image for each stage shows the overlay of CBP4a (red), α-tubulin (green), and DAPI (blue). At least four independent replicates were performed, all of which showed the same result. Scale bar represents 2 μm.

CBP4a also redistributed during mitosis, but with a very different pattern of redistribution from FhkA. Unlike FhkA, which was detected throughout the entire cell, CBP4a redistributed exclusively within the nucleoplasm. During prometaphase CBP4a was detected as several discrete, spots which were distributed throughout the nucleoplasm hereafter referred to as “CBP4a islands” (Figure 6). During metaphase these islands were still detected along with two larger islands that localized to the metaphase plate region. During anaphase and telophase these islands were mostly detected along the inner nuclear membrane. In fact during telophase the islands were distributed in an arrangement reminiscent of reforming nucleoli (Figure 6).

### Nucleolar localization of CBP4a is Ca^2+^-dependent

**Figure 7:**
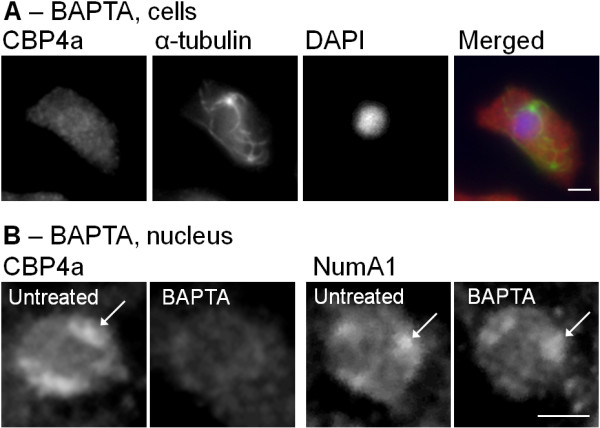
**Nucleolar localization of CBP4a is Ca2**^**+−**^**dependent. (A)** Immunolocalization of CBP4a in BAPTA-treated cells. Immunolocalization of α-tubulin was used to verify cellular integrity and DAPI was used to identify the nucleus. A merged image shows the overlay of CBP4a (red), α-tubulin (green), and DAPI (blue). **(B)** Immunolocalization of CBP4a or NumA1 in the nucleus of either untreated or BAPTA-treated cells. Arrows denote nucleoli. Scale bar represents 2 μm. At least four independent replicates were performed, all of which showed the same result.

Given that CBP4a interacts Ca^2+^-dependently with nucleolar NumA1 we were interested to determine if Ca^2+^ levels affected its localization. Treatment with 5 mM of the Ca^2+^ chelator BAPTA (4 hours) resulted in the disappearance of nucleolar CBP4a (Figure 7A, B). In contrast, BAPTA did not affect the localization of NumA1 as previously documented (Figure 7B) [24]. Localization of CBP4a was not affected by either 1, 5, or 10 mM CaCl_2_ (4 hours) or the Ca^2+^ chelator EGTA (1, 5, or 10 mM for 4 hours). It is important to note that BAPTA chelates Ca^2+^ with greater affinity than EGTA. As well, since NumA1 is a CaM-binding protein it was possible that CaM could indirectly affect CBP4a localization via interactions with NumA1. However CaM antagonists (300 μM W-5 or 50 μM W-7 for 4 hours) had no effect on the localization of CBP4a.

## Discussion

Here we provide novel insight into two new nucleolar proteins, their localization and their translocations during mitosis. FhkA and CBP4a are both nucleolar proteins which distribute differentially during mitosis. Prior to this study only two *Dictyostelium* nucleolar proteins had been investigated during mitosis: NumA1 and Snf12 [24,25]. Combined with previous studies, a greater understanding of nucleolar structure and nucleolar dynamics is being elucidated.

During interphase, FhkA localized around the nucleolar periphery in a pattern similar to *Dictyostelium* heat shock protein 32 (Hsp32) as revealed by Moerman and Klein [26]. However unlike FhkA, Hsp32 localizes in the shape of a beaded string around the periphery of each nucleolar patch, a pattern corresponding to the distribution of nucleolar rDNA in this organism [26,51]. Fittingly, Hsp32 has been shown to interact with rDNA in spite of this interaction not being responsible for its localization [26]. One difference between the localization of FhkA and Hsp32 is that FhkA did not appear as a beaded string and it was detected predominantly on the side adjacent to the nuclear envelope rather than around the full periphery of the nucleolar patch.

CBP4a localized to these nucleolar patches during interphase in a pattern similar to that described previously for eif6, NumA1, and Snf12 [22,24,25]. Although EGTA treatment did not affect the localization of CBP4a, treatment with BAPTA (which chelates Ca^2+^ with more specificity than EGTA) resulted in the disappearance of these patches demonstrating that the nucleolar localization of CBP4a is Ca^2+^-dependent. In contrast, BAPTA treatment did not affect the localization of nucleolar NumA1 fitting with previous results [24]. These results suggest that CBP4a is recruited to the nucleolus by NumA1 in a Ca^2+^-dependent manner as previously suggested by Myre and O’Day [36]. Unlike CBP4a, NumA1-binding partner PsaA colocalizes with NumA1 in the nucleoplasm but not the nucleolus [34,35].

It is possible that anti-CBP4a may also detect CBP4b since the two evolved on a separate branch from the majority of the other CBPs in *Dictyostelium*. Previous data is insufficient to determine any potential differences in function or localization between them [39]. Although the region in CBP4b most resembling the antigen site in CBP4a differs only by one residue (CBP4b contains A instead of G at position 26) analysis of mRNA expression revealed that in vegetative cells CBP4a mRNA is approximately 8.3-times more abundant that that of CBP4b suggesting that any detection of CBP4b by anti-CBP4a would be minimal [52,53].

AM-D treatment results in nucleolar dissolution accompanied by the disappearance of nucleolar proteins in *Dictyostelium* which is consistent with observations by others [20,22,24]. CBP4a also disappeared from the nucleolus after AM-D treatment. In contrast, FhkA protruded from the nucleus to eventually pinch off as cytoplasmic circles. Given the relationship between microtubules and the nucleolar protrusion that occurs during the aggregation stage of *Dictyostelium* development we decided to investigate whether microtubules also functioned during the AM-D-induced protrusion observed here. However the AM-D-induced protrusions were still observed despite the lack of microtubules suggesting that this mechanism of nucleolar protrusion is fundamentally different than that observed during aggregation that occurs during normal multicellular development. Interestingly, the same AM-D-induced phenomenon of nuclear protrusion was also observed for nucleolar Snf12 but not for NumA1 or eif6 [22,24,25]. In all cases, the location of these protrusions was on the side of the nucleolus farthest from the centrosome which was the same as for Snf12 [25].

It is interesting to note that in yeast nuclear extensions are observed during mitotic delay [54]. These extensions occur in the nuclear envelope adjacent to the nucleolus and are thought to serve as a membrane sink – a way to store an expanding nuclear membrane while maintaining a functional nucleus. The morphology of these nuclear extensions varies greatly compared to the relatively uniform budding observed for FhkA, CBP4a, and Snf12 and it is not known if in yeast these extensions eventually separate from the nucleus, as is the case in *Dictyostelium*. Nevertheless, the nucleolar budding observed in *Dictyostelium* may represent a similar phenomenon perhaps in response to the AM-D-induced mitotic arrest. Perhaps these buds separate from the nucleus in order for the membrane to be degraded in the cytoplasm. Proteins that accumulate in these buds/extensions may represent an excess of proteins produced for mitosis which during mitotic arrest would no longer be needed and thus destined for degradation.

**Figure 8:**
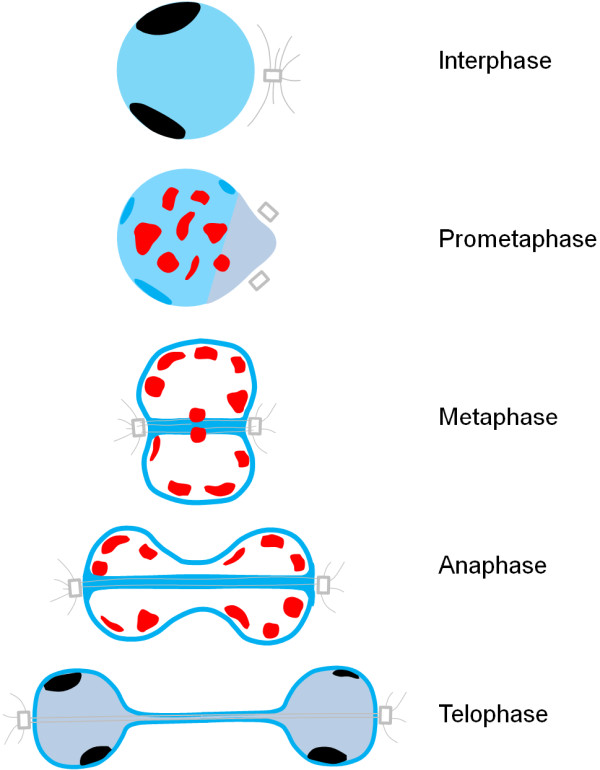
**Binding partners CBP4a and NumA1 localize to different intranuclear subdomains during mitosis in *Dictyostelium*.** During interphase CBP4a and NumA1 colocalize in the nucleolus (black) while NumA1 also shows nucleoplasmic localization to a lesser extent. During prometaphase, CBP4a (red) reorganizes as nucleoplasmic islands distributed throughout the nucleoplasm while NumA1 (blue) remains at the nuclear periphery but in smaller patches. During metaphase the CBP4a islands localize around the periphery of the nucleoplasm as well as the metaphase plate while NumA1 localizes at the nuclear envelope region and along spindle fibres. This pattern remains constant during anaphase with the exception of the disappearance of the CBP4a islands at the metaphase plate region. During telophase the CBP4a islands begin to enlarge while NumA1 reappears in nucleoli and the nucleoplasm but is still present along the nuclear envelope region. It is not clear if the large CBP4a islands during telophase colocalize with NumA1 in reforming nucleoli [24].

In most species nucleolar proteins redistribute to non-nucleolar locales during mitosis [9-12]. This is also the case in *Dictyostelium*[24,25]. Nucleolar protein dynamics during mitosis have now been studied for four proteins: NumA1, Snf12, FhkA, and CBP4a. NumA1 and CBP4a offer unique insight into nuclear and nucleolar dynamics during *Dictyostelium* mitosis as they remain intranuclear during this time. A diagrammatic interpretation of the nuclear and nucleolar dynamics of NumA1 and CBP4a during karyokinesis is presented in Figure 8. During mitosis, NumA1 distributes diffusely in the nucleoplasm as well as at the nuclear periphery, in the centrosomal region, and at the spindle fibre region [24]. In contrast, its binding partner CBP4a redistributes to multiple “CBP4a islands” within the nucleoplasm during mitosis and as two discrete “CBP4a islands” at the metaphase plate region during metaphase. Whether these two sets of islands are organized in the same way and whether they represent a specific nucleoplasmic region that arises during mitosis remains to be validated. The fact that NumA1 and CBP4a do colocalize in the nucleolus during interphase but redistribute to discrete and different locations during mitosis suggests that their Ca^2+^-dependent interaction is related to a specific co-function in the nucleolus during interphase. The distinct localization patterns of these proteins suggest there could be intranuclear subdomains linked to mitosis that have not been previously identified in *Dictyostelium*.

In contrast to CBP4a, FhkA redistributed throughout the entire cell during mitosis in a pattern similar to that of Snf12 [25]. It was also detected at the nuclear envelope region from prometaphase through telophase. This mitotic redistribution pattern was also similar to that of nuclear PsaA and cdk5, two recently identified cell cycle-linked proteins in *Dictyostelium*[34,35,42]. It was not possible to determine if FhkA was adjacent to the inner nuclear envelope, outer nuclear envelope, or colocalized with the nuclear envelope, however the pattern was similar to that observed for NumA1. Thus, NumA1, CBP4a, Snf12, and FhkA represent the first nucleolar proteins for which their dynamics during mitosis have been studied.

## Conclusions

We have identified two novel nucleolar proteins in *Dictyostelium*, FhkA and CBP4a. CBP4a localizes Ca^2+^-dependently throughout the nucleolus during interphase while FhkA resides at the nucleolar periphery. During mitosis FhkA redistributes throughout the entire cytoplasm while CBP4a relocates to intranuclear “CBP4a islands”, perhaps representing previously unidentified nuclear subdomains. The intranuclear redistribution of CBP4a during mitosis is different than that previously observed for binding partner NumA1, which instead localizes predominately to the nuclear periphery. FhkA and CBP4a represent only the third and fourth nucleolar proteins to be studied during mitosis in *Dictyostelium* but nevertheless shed new light on the dynamic structural changes taking place within the nucleus during this time.

## Methods

### Polyclonal antibody production

Two polyclonal antibodies were produced in rabbits. A peptide equivalent to a unique C-terminal sequence from FhkA (^554^NQLQLNEGELLKKRKTFLSN^573^; Figure 1) and one equivalent to residues ^20^KAGDKNGDNQLSKKE^34^ from CBP4a were synthesized using solid phase synthesis (with the addition of an N-terminal cysteine required for subsequent conjugation), purified via HPLC, and verified via mass spectroscopy prior to KLH conjugation via an MBS linker (Advanced SynTech Inc.). Conjugation was performed as previously described [55]. Polyclonal antibodies were produced in New Zealand White rabbits against this KLH-conjugated peptide according to the standard operating procedures of the University of Toronto. Rabbits were injected with 750 μg of KLH-conjugated peptide in 500 μL PBS mixed with 500 μL Freund’s Complete Adjuvant (Sigma-Aldrich®). Three subsequent boost injections 2–3 weeks apart were performed in the same manner each consisting of 250 μg of KLH-conjugated peptide in 500 μL PBS mixed with 500 μL Freund’s Incomplete Adjuvant (Sigma-Aldrich®). Western blots were performed with serum from blood samples collected prior to exsanguination as previously described [49]. Exsanguination was performed by the department of Faculty of Medicine at the University of Toronto and the serum was collected. Crude serum was used for immunolocalization experiments and referred to as either anti-CBP4a or anti-FhkA.

### SDS-PAGE and western blotting

Cells (1–2 × 10^6^ cells/mL) in HL-5 were resuspended in lysis buffer (50 mM Tris pH 8.0, 150 mM NaCl, 0.5% NP-40, 1 complete protease inhibitor tablet; Roche Diagnostics) and sonicated. Protein levels were quantified using the Bradford assay. 20 μg protein were separated on a 12% SDS-PAGE gel. Proteins were then transferred onto a PVDF membrane (Pall corp.). The membrane was blocked with 5% non-fat milk in washing buffer (TBS and 0.001% Tween 20) at 4°C overnight and subsequently probed with anti-FhkA (Santa Cruz®) in 5% non-fat milk in washing buffer (1:40) for one hour at room temperature followed by probing with secondary anti-mouse HRP (1:600; Santa Cruz®) in the same conditions. Bands were detected with Amersham ECL Plus^TM^ Western Blotting Detection kit (General Electric) using the STORM Scanner System.

### Treatment, fixation with ultracold methanol, immunolocalization, and live viewing

*Dictyostelium* AX3 cells were grown in HL-5 at 21°C shaking at 180 rpm, as previously described [56]. For fixation and immunolocalization experiments cells (150 μL of 2 × 10^6^ cells/mL) were allowed to adhere to 0.1 mm circular glass cover slips (McCrone™) for 30 minutes in a humidity chamber. Alternatively, cells were treated while adhering to coverslips (in HL-5) with either 1, 5, or 10 mM CaCl_2_ (4 hours), 1, 5, or 10 mM EGTA (4 hours), 5 mM BAPTA (4 hours), 300 μM W-5 (4 hours), 50 μM W-7 (4 hours), 50 μM nocodazole (6 hours), or 0.05 mg/mL actinomycin-D (AM-D; 4 hours, 8 hours, or 4 hours followed by three washes with HL-5 prior to 4 hours recovery in HL-5). Cells were fixed with ultracold methanol as previously described [57]. After blocking, cells were incubated with either anti-CBP4a or anti-FhkA for 60 minutes followed by Alexa Fluor® 555 goat anti-rabbit (Invitrogen™) for 45 minutes. Cells were then incubated with anti-α-tubulin (Hybridoma Bank) for 60 minutes followed by Alexa Fluor® 488 goat anti-mouse (Invitrogen™) for 45 minutes. The pattern of microtubules was used to determine cell cycle stage. ProlongAntifade (Invitrogen™) containing DAPI (5 μL) was placed on a slide prior to mounting and cover slips were then sealed using nail polish. Cells were viewed with a Nikon 50i epifluorescent microscope equipped with a Nikon Digital-Sight DS-Ri1 camera and images were analyzed using NIS Elements BR 3.0.

## Abbreviations

AM-D: Actinomycin-D; CaM: Calmodulin; CBP: Ca^2+^-binding protein; CBP4a: Ca^2+^-binding protein 4a; Eif6: Eukaryotic translation initiation factor 6; FhkA: Forkhead-associated kinase A; FHA: Forkhead-associated; Hsp32: Heat-shock protein 32; PsaA: Puromycin-sensitive aminopeptidase A.

## Competing interests

The authors declare that they have no competing interests.

## Authors’ contributions

AC performed the experiments, participated in the conception and design of the study, participated in analysis and interpretation of the data, and drafted/edited the manuscript. DO participated in the conception and design of the study, participated in analysis and interpretation of the data, and revised/edited the manuscript. All authors read and approved the final manuscript.
